# Proprotein Convertase Subtilisin/Kexin Type 9 Promotes Gastric Cancer Metastasis and Suppresses Apoptosis by Facilitating MAPK Signaling Pathway Through HSP70 Up-Regulation

**DOI:** 10.3389/fonc.2020.609663

**Published:** 2021-01-07

**Authors:** Beili Xu, Shuyu Li, Yong Fang, Yanting Zou, Dongqiang Song, Shuncai Zhang, Yu Cai

**Affiliations:** ^1^ Department of Gastroenterology and Hepatology, Zhongshan Hospital, Fudan University, Shanghai, China; ^2^ Department of General Surgery, Zhongshan Hospital, Fudan University, Shanghai, China; ^3^ Department of Hepatic Oncology, Zhongshan Hospital, Fudan University, Shanghai, China

**Keywords:** proprotein convertase subtilisin/kexin type 9, gastric cancer, heat shock protein 70, MAPK pathway, prognosis

## Abstract

**Objective:**

To examine the effect of proprotein convertase subtilisin/kexin type 9 (PCSK9) on gastric cancer (GC) progression and prognosis, and to explore the underlying mechanism.

**Methods:**

PCSK9 expression levels in human GC tissues were determined by quantitative real-time PCR, western blotting, and immunohistochemical assay. PCSK9 serum levels were detected by enzyme-linked immunosorbent assay. The relationships of PCSK9 and GC progression and survival were analyzed using the Chi-square test, Kaplan-Meier analysis, and Cox proportional hazards model. The effect of PCSK9 on cell invasion, migration, and apoptosis were determined in human GC cell lines and mouse xenograft model separately using PCSK9 knockdown and overexpression strategies. The PCSK9 interacting molecules, screened by co-immunoprecipitation combined with LC-MS/MS, were identified by immunofluorescence localization and western blotting. Additionally, the mitogen-activated protein kinase (MAPK) pathway was assessed by western blotting.

**Results:**

PCSK9 mRNA and protein levels were significantly elevated in GC tissues compared with the paired normal tissues at our medical center (P < 0.001). Notably, the up-regulation of PCSK9 expression in GC tissues was related to tumor progression and poor survival. GC patients had higher serum levels of PCSK9 than the age-matched healthy controls (P < 0.001); PCSK9 promoted invasive and migratory ability and inhibited apoptosis in GC cells with no apparent affection in cell proliferation. The silencing of PCSK9 reversed these effects, suppressing tumor metastasis *in vitro* and *in vivo*. Furthermore, PCSK9 maintained these functions through up-regulating heat shock protein 70 (HSP70), ultimately facilitating the mitogen-activated protein kinase (MAPK) pathway.

**Conclusion:**

Collectively, our data revealed that high PCSK9 expression levels in GC tissue were correlated with GC progression and poor prognosis and that PCSK9 could promote GC metastasis and suppress apoptosis by facilitating MAPK signaling pathway through HSP70 up-regulation. PCSK9 may represent a novel potential therapeutic target in GC.

## Introduction

Gastric cancer (GC) is one of the most lethal carcinomas in the world, which ranks fifth in incidence and the third in the leading cause of cancer-related death (1). Worldwide over one million new cases and approximately 783,000 deaths were reported in 2018 (2). Despite the gradually declining incidence in Northern America and Europe, it is worth noting that GC still remains a high incidence and mortality in Eastern Asia (2–4). The prognosis of patients with advanced GC remains unsatisfactory (5). Patients often progress to a serious stage by the time they seek medical attention. Therefore, it is imperative that potential biomarkers be identified to better understand the development and progression of GC and develop new strategies to improve the prognosis of GC patients.

Pro-protein convertases (PCs) are a group of Ca^2+^-dependent secretory mammalian serine proteases (6). Structurally, the PCs start from the signal peptide at the N-terminal extremity and end with the specific C-terminal domain; adjacent to both ends are the pro-domain that keep the enzyme inactive, and the P-domain regulated by calcium and PH, and in the middle is the high homology catalytic domain, the particular structure making the PCs exhibit highly selective cleavage rule at the basic residues, and after cleavage, the inactive precursors become bioactive proteins and peptides, the bioactive peptides playing a variety of biological functions in viral infections, auto-immune diseases, metabolic diseases, and malignant tumors (7).

As a unique member of the proprotein convertase family, proprotein convertase subtilisin/kexin type 9 (PCSK9) can auto-catalytically process its pro-segment in the absence of basic or non-basic amino acid (8–10). Previous studies have proved that PCSK9 plays an important role in cholesterol metabolism and the treatment of coronary atherosclerosis (11–13). A growing number of recent studies have found that PCSK9 becomes a latent tumor-targeting molecule which has the value of potential application in a variety of tumors (14–16). In 2013, PCSK9 was reported to be identified as an excellent biomarker for the early detection of gastric adenocarcinoma (17). It can be successfully screened by a SILAC-based quantitative proteomic approach and validated by immunohistochemical labeling. However, little is known about PCSK9 as an oncogene in GC. Hence, our current study aimed to investigate the role of PCSK9 in GC and its underlying mechanism.

## Materials and Methods

### Patients Information and Tissue Samples

A total of 155 gastric adenocarcinoma patients were consecutively enrolled in the study between January 2012 and December 2016, who would be followed up until December 2018. GC specimens and corresponding adjacent normal tissues were collected at the time when the patients underwent gastroscopy or surgical resection in Zhongshan Hospital of Fudan University (Shanghai, China). Lymph node metastasis was verified by postoperative pathology. None of the patients had received radiotherapy or chemotherapy before operation. The blood samples were collected before surgery to be centrifuged at 2000 rpm for 10 min to separate serum, which was to be stored in -80°C refrigerator for later use. A collection was made of the clinicopathologic characteristics of all patients such as age, gender, tumor size and differentiation, Lauren’s classification, TNM stage, and prognosis (**Supplementary Table 1**). TNM stage was assessed according to the American Joint Committee on Cancer (AJCC) 8^th^ edition of GC (18). Thirty age-matched controls were composed of healthy volunteers who were required to undergo a regular medical examination without taking statins or PCSK9 antibody.

### Reagents and Antibodies

TRC051384 (termed as heat shock protein 70(HSP70) agonist, catalog no. HY-101712) and apoptozole (termed as HSP70 inhibitor; catalog no. HY-15098) were purchased from MedChemExpress (New Jersey, USA). Rabbit monoclonal antibodies against PCSK9 (catalog no. 85813) for western blotting, HSP70(catalog no. 4873), p38 (catalog no. 8690), phosphorylated (p)−p38(catalog no. 9215), extracellular signal−regulated kinase 1/2 (ERK1/2; catalog no. 4695), p−ERK1/2 (catalog no. 4370), c−Jun N−terminal kinase (JNK; catalog no. 9252), and p−JNK (catalog no. 9251) were acquired from Cell Signaling Technology (Danvers, MA, USA). Rabbit monoclonal antibody against PCSK9 (catalog no. ab28770) for immunohistochemistry, immunoprecipitation and immunofluorescence was purchased from Abcam (Cambridge, MA, USA). Mouse monoclonal antibody against β−actin (AA128) was purchased from Biotime Biotechnology Co. Ltd. (Shanghai, China).

### Cell Lines and Culture

Human GC cell lines (SGC-7901, MKN-45, MKN-28, KATO-III, AGS, MGC-803 and NCI-N87) and human gastric mucosal epithelial cell line (GES-1) were purchased from Shanghai Institute of Biochemistry and Cell Biology, Chinese Academy of Sciences (Shanghai, China). AGS cells were cultured in DMEM/F12 medium (Hyclone, Logan, UT, USA), and NCI-N87 cells, in RPMI-1640 medium (Hyclone, Logan, UT, USA) and others, in IMDM medium (Hyclone, Logan, UT, USA), containing10% fetal bovine serum (FBS, Gibco, Carlsbad, CA, USA), 100 μg/ml of streptomycin and 100 U/ml penicillin in an humidified air atmosphere incubator with 5% CO_2_ at 37°C.

### Plasmids and Transfection

The short hairpin(sh) RNAs against PCSK9 and negative control shRNA were synthesized by Genomeditech (Shanghai, China) and were inserted into pGMLV-SB7 cloning vector (Genomeditech, Shanghai, China). The coding regions of human PCSK9 was cloned into the expression vector pGMLV-CMV-MCS-PGK-Puro (Genomeditech, Shanghai, China). The empty vector was packaged as negative controls.

SGC-7901 and MGC-803 cells were infected with the lentivirus-transducing units according to the manufacturer’s protocol. The stable transfectants were selected and cultured in medium containing blasticidin or puromycin separately. The efficiency of genetic silencing or overexpression was evaluated by western blotting. We selected PCSK9 shRNA‐1 with the highest efficiency for the further mechanism study.

### RNA Extraction and Quantitative Real-Time PCR

Total RNA was isolated from the frozen tissues or harvested cells with RNA extraction kit (Promega, Madison, Wi, USA) and reversed to cDNA using GoScript™ Reverse Transcription Mix, Random Primers (Promega, Madison, Wi, USA). The cDNAs were amplified by Eastep^®^ qPCR Master Mix Kit (Promega, Madison, Wi, USA) on ABI Prism 7500 Sequence Detection system (Applied Biosystems, Tokyo, Japan). The relative target gene expression was analyzed using 2-△△Ct method, and β-actin was used for normalization. The primer sequences were used as follows: human PCSK9 (forward:5’-CACGGAACCACAGCCACCTT-3’; reverse: 5’-CGCCACTCATCTTCACCAGGAA-3’; β-actin (forward: 5’-AATCTGGCACCACACCTTCTA-3’; reverse: 5’-ATAGCACAGCCTGGATAGCAA-3’).

### Western Blotting

Proteins were isolated from the cells and tissues using RIPA lysis buffer (Biotime, Shanghai, China). The protein concentration was measured with BCA enhanced Protein Assay Kit (Biotime, Shanghai, China). The total proteins were loaded onto SDS-PAGE gels (Biotime, Shanghai, China). After electrophoresis, the proteins were transferred to polyvinylidene fluoride (PVDF) membrane (Millipore, Billerica, MA, USA), which was to be incubated with primary antibodies. After incubated with horseradish peroxidase-conjugated secondary antibodies, the immune complexes were detected with ECL Detection Kit (Millipore, Billerica, MA, USA) and quantified using Gel-Pro Analyzer (Media Cybernetics Corporation, USA).

### Immunohistochemistry

The histological sections of gastric tissues were deparaffinized and rehydrated before subjected to heat-induced antigen retrieval. After incubated with primary antibodies overnight at 4°C, the secondary antibody was incubated at room temperature for 1 hr. Upon DAB substrate color rendering, the histological sections were sealed using neutral gum to be photographed. The expression level was independently evaluated by 2 senior pathologists according to the intensity and proportion of positive cells. The staining intensity was graded on a four-point scale: 0, negative; 1, weak; 2, intermediate; and 3, strong, and the percentage of positive cells was divided into five grades: 0, 0%; 1, 1–25%; 2, 26–50%; 3, 51–75%; and 4, 76–100%. The final score was calculated by multiplying the intensity and percentage scores. An overall score of 0–12 was acquired and graded as: score 0, negative; score 1–4, weak; score 5–8, moderate; and score 9–12, strong.

### Enzyme-Linked Immunosorbent Assay

The serum levels of PCSK9 were detected using an ELISA Kit (Abcam, Cambridge, UK), and the assay was performed according to manufacturer’s instructions. In short, 50 μl per well of serum and standard solution was added to the plates, followed by an addition of 50 μl of the Antibody Cocktail specific for PCSK9 before incubated for 1hr at room temperature. Afterward, the plates were washed, and an addition of TMB development solution was made to generate blue coloration. With sulfuric acid added to stop the reaction, the absorbance was measured at a wavelength of 450nm using a microplate reader (FlexStation 3, Molecular Devices, USA).

### Clonogenic Assay

GC cells were seeded at a density of 1 × 10^3^ cells/well in a six-well plate. After culturing for 15 days, the cells were fixed by adding an appropriate amount of methanol. Afterward, the cells were stained with 0.1% crystal violet solution (Biotime, Shanghai, China) and photographed. The number of colonies containing more than 50 cells was counted manually.

### Cell Counting Kit-8 (CCK-8) Assay

When plated in 96-well plates at 2 × 10^3^ cells per well, SGC-7901 and MGC-803 cells were cultured with Cell Counting Kit (CCK8, Biotime, Shanghai, China) for 24, 48, 72, 96, and 120 h, respectively. Absorbance at 450nm was measured with the microplate reader (FlexStation 3, Molecular Devices, USA).

### Scratching Assay

Parallel lines were drawn using a marker on the floor of the 6-well plate. The cells were seeded in 60-mm culture wells at 5 × 10^5^ cells/well. An artificial “wound” was created with a 200 μl pipette tip. The wound width was recorded and measured at the two time points of 0 and 24 h after scratching under an optical microscope (Olympus, Tokyo, Japan) to evaluate the migration of the tested cells.

### Transwell Migration and Invasion Assay

The migration and invasion of GC cells were determined using 24-well Transwell chambers (Corning, NY, USA) coated without or with Matrigel (Corning, NY, USA). The cells were harvested to be suspended in the serum-free medium supplemented with 1% BSA. In the migration test,100 μl of solution containing approximately 1х10^5^ cells was plated into the upper compartment, and 600 μl of 10% FBS containing medium was added to the bottom compartment. Following a 24h incubation at 37°C, the cells on the upper-side of the membrane were removed with clean swabs. Fixed with 4% paraformaldehyde, the samples were stained using crystal violet (Biotime, Shanghai, China). The number of the cells was counted and photographed under an optical microscopy (IX51, Olympus, Japan). In the invasion assay, 50μg Matrigel and 45 μl of serum-free medium were spread on each membrane before incubated at 37°C overnight to solidify the Matrigel. Into the upper compartment were plated 100 μl of the cells. The remaining procedures were identical to those in the cell migration test.

### Cell Apoptosis Assay

The cell apoptosis was detected with the flow cytometry method. The cells were collected and washed in cold PBS before centrifuged at 1000rpm for 5 min. After resuspended in 100 μl of binding buffer, the cells were labeled with 5 μl of FITC Annexin V and 5 μl of propidium iodide (BD Pharmingen™, USA) for 15 min at room temperature in the dark. Terminally, 400 μl of binding buffer was added to each tube and a FACS Aria II (BD) was used to evaluate the apoptotic cells.

### Co-Immunoprecipitation (co-ip) and LC-MS/MS Analysis

Cells were lysed with the cell lysis buffer supplemented with complete protease inhibitor cocktail (Roche, Switzerland) and the proteins were pre-cleared. Protein-G-A agarose beads (Roche, Switzerland) were incubated with anti-PCSK9 antibody or non-specific rabbit IgG antibody (CST, Danvers, MA, USA) at 4°C overnight. Becoming pelleted, the precipitates were washed thrice with the lysis buffer, before analyzed using western blotting. After electrophoresis, the gels were stained using Coomassie Blue Fast Staining Solution (Biotime, Shanghai, China) and a Q-Exactive mass spectrometer (Thermo Scientific, USA) was used to do the LC-MS/MS analysis.

### Immunofluorescence Staining

Sterilized glass coverslips were put onto a 24-well plate, followed by an addition of the cell suspension with 50% seeding density, before incubated overnight. After15-min fixing with 4% paraformaldehyde at room temperature, 0.3% Triton X-100 (Biotime, Shanghai, China) was added to break the cell membrane. Successively were added primary antibody, corresponding secondary antibody and DAPI according to the protocol. In the end, one drop of mounting medium containing anti-fluorescence quencher was placed onto each slide. Then the coverslip was picked up, inverted onto drop in a cell-side-down manner and nail-polish-sealed. Images were collected under a confocal fluorescence microscope (FV3000, Olympus).

### Xenograft Tumor Assay

Thirty BABL/c nude mice aged 4 weeks, purchased from Slack Laboratory Animal Co., ltd (Shanghai, China), were kept under the specific pathogen-free (SPF) condition, and maintained in a climate-controlled room (12:12h artificial dark-light cycle with a stable room temperature of 23 ± 2°C), gaining free access to standard rodent chow and water. After that, the mice were equally divided into three groups at random: PCSK9 shRNA with/without HSP70 agonist, and PCSK9 shNC group, respectively. A total of 1 × 10^6^ SGC-7901 cells with/without PCSK9 shRNA lentivirus were digested and suspended in 100 μl of bacterial-free PBS. Each group was injected with relevant cells *via* tail vein according to the preimplantation experiment. The next day those which bore PCSK9 shRNA SGC-7901 cells were treated with physiological saline or TRC051384 intraperitoneally at a dose of 9 mg/kg as first dose and then 4.5 mg/kg every 12 h thereafter for 10 days, as previously described (19). Four weeks later, the mice were subjected to^18^F-FDG positron emission tomography (PET) scan (MedicLab PET/MR, Madic Technology Co. Ltd, Shandong, China) to show a rough picture of tumor metastasis. Five weeks after SGC-7901 injection, the mice were sacrificed, the lungs isolated, photographed and fixed in formalin. Afterward, the lung tissues were embedded in paraffin to be cut into 4-μm segments, which were to be stained using the standard hematoxylin and eosin (H&E) staining method for later metastatic nodules calculation.

### Statistical Analysis

Statistical analyses were performed using SPSS 20.0 software (SPSS, Chicago, IL, USA) and GraphPad Prism8.0 (GraphPad Software, Inc., San Diego, CA, USA), the data shown as mean ± S.D. or Median (Q1-Q3) according to the normal distribution test. The significance of the differences was analyzed with Student’s t-test, one-way analysis of variance (ANOVA) or Mann-Whitney U-test, and the relationship between clinicopathological factors and PCSK9 expression, with the chi-square test. The diagnostic value of PCSK9 expression in GC was measured using the receiver operating characteristics (ROC) curve. The survival analysis was assessed by the Kaplan-Meier method and examined by the log-rank test. Univariate and multivariate analyses were performed using the Cox proportional hazards regression model. P < 0.05 was considered to be statistically significant.

## Results

### Increased PCSK9 Expression in Primary GC Tissues and Its Overexpression associated With Lymph Node Metastasis and Poor Prognosis

#### PCSK9 was Upregulated in Primary GC Tissues

**Figure 1:**
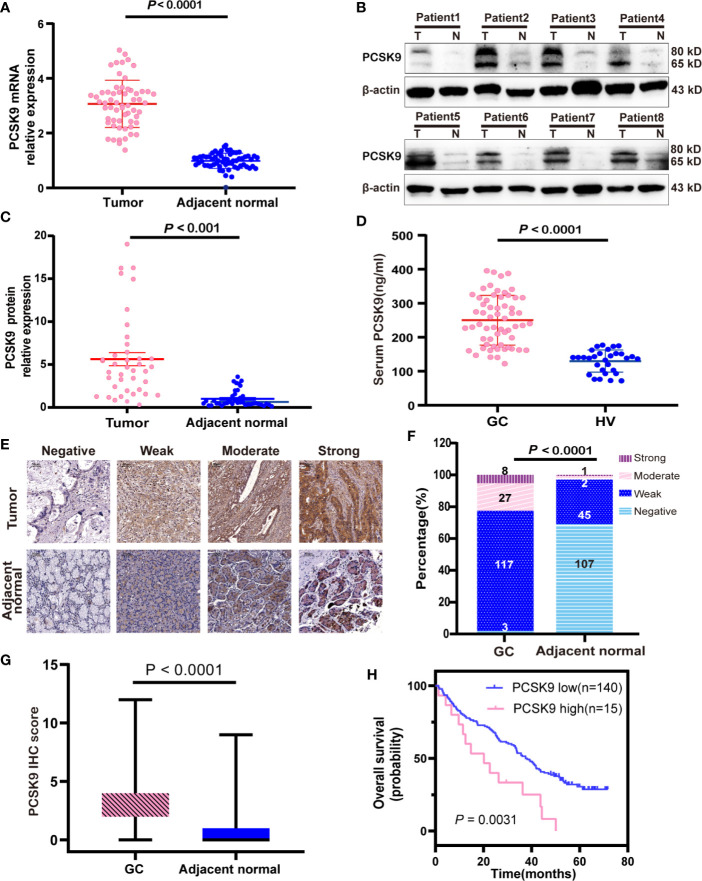
Increase of PCSK9 expression in GC. **(A)** PCSK9 mRNA expression levels assessed in paired GC and adjacent normal tissues (P < 0.0001; n = 60) **(B)** PCSK9 protein expression levels measured in GC tissues and related adjacent non-tumorous tissues in representative eight patients by western blotting; β-actin served as the internal control. **(C)** Comparison of relative PCSK9 protein expression in 40 GC tissues and adjacent non-tumorous tissues by western blotting (P < 0.001). **(D)** Serum levels of PCSK9 analyzed by ELISA in GC patients (n = 60) and healthy volunteers (HV; n = 30; P < 0.0001). **(E)** Representative IHC image of PCSK9 protein in GC and adjacent normal tissues. **(F)** Distribution of PCSK9 IHC score in GC and adjacent normal tissues (P < 0001). **(G)** IHC score in GC and adjacent normal tissues (n = 155; P < 0.001). **(H)** Overall survival curve for GC patients with high vs. low expression of PCSK9 IHC score generated with Kaplan-Meier methods (P < 0.01).

To explore the expression of PCSK9 in GC, we first evaluated the mRNA and protein expression levels of PCSK9 in paired GC and adjacent non-tumor tissues by qRT-PCR (n = 60; **Figure 1A**) and western blotting (n = 40; **Figures 1B, C**), the results showing that PCSK9 expression was significantly higher at both the transcriptional and protein level in the tumor tissues than in the adjacent normal tissues (P < 0.0001).

#### Increased PCSK9 Levels in the Peripheral Blood of GC Patients

The increased PCSK9 expression in GC tissues led to the investigation of PCSK9 serum levels in the GC patients, which through ELISA detected serum PCSK9 levels in 60 GC patients and 30 age-matched healthy controls. The results showed that PCSK9 serum levels were significantly higher in the GC patients than in the controls (250.1 ± 73.20 vs. 129.9 ± 32.75 ng/ml; P < 0.0001; **Figure 1D**).

#### Overexpression of PCSK9 in GC Tissues was Associated With Poor Prognosis and Lymph Node Metastasis

In order to clarify the clinical significance of PCSK9, immunohistochemical (IHC) staining was performed and PCSK9 expression was scored in 155 GC tissues and their matched adjacent non-cancerous tissues. The results indicated that PCSK9 was mostly localized to the cytoplasm and partially to the extracellular matrix in GC tissues; and that PCSK9 was distinctly enhanced in GC tissues when compared with their matched adjacent non-cancerous ones (**Figures 1E–G**.). In the GC cohort, 8 patients (5.16%) had strong PCSK9 staining, 27 (17.42%) had the moderate, and 117 (75.48%) had the weak, respectively. Only 3 patients (1.94%) produced no positive PCSK9 expression. As indicated by the ROC curve to define low and high PCSK9 expression level, the cutoff point was found as 7.5, and the area under a curve (AUC) was 0.6274 (95% CI: 0.4997–0.7550). To this end, IHC score during 8–12 was determined as high PCSK9 expression, whereas the others were determined as low.

A collection was made of the clinicopathological characteristics of all the 155 patients for the chi-square test to explore the correlation between PCSK9 expression and the values. As shown in **Table 1**, the high expression of PCSK9 in GC was significantly correlated with lymph node metastasis (P < 0.05). However, no statistically significant correlations were observed between PCSK9 and other clinicopathological variables such as age (P = 0.609), sex (P = 0.7), tumor differentiation (P = 0.578), Lauren’s classification (P = 0.574), tumor size (P = 0.204), tumor depth of infiltration (P = 0.647), and TNM staging (P = 0.304).

**Table 1:** PCSK9 expression levels and clinicopathologic characteristics in GC patients.

Variables	N	PCSK9 expression		χ^2^	P
		Low (n = 140)	High (n = 15)		
Sex					
Male	100	91	9	0.148	0.700
Female	55	49	6		
Age, y					
≤60	45	42	3	0.262	0.609
>60	110	98	12		
Tumor differentiation					
Well and moderately	52	46	6	0.310	0.578
Poorly	103	94	9		
Tumor size (cm)					
<5	101	89	12	1.611	0.204
≥5	54	51	3		
Lauren’s classification					
Intestinal type	72	64	8	0.316	0.574
Diffuse and mixed type	83	76	7		
T stage					
I-II	20	17	3	0.209	0.647
III-IV	135	123	12		
Lymph node metastasis					
N1-3	117	102	15		**0.023^#^**
N0	38	38	0		
M stage					
M1	5	4	1	0.001	0.980
M0	150	136	14		
TNM stage					
I-II	49	42	7	1.055	0.304
III-IV	106	98	8		

^#^Fisher’s exact test. Bolded text shows P<0.05.

Next, we investigated the relationship between PCSK9 expression and prognostic outcome in the GC patients, whose follow-up period ranged from 1.3 to 71.7 months, with a median overall survival time of 35.6 months. The survival time was compared between the patients with low PCSK9 expression (n = 140) and those with high PCSK9 expression (n = 15) by Kaplan-Meier survival analysis, the result showing that high PCSK9 expression predicted a worse outcome in the GC patients (P < 0.01; **Figure 1H**). As indicated by the univariate COX regression model, Lauren’s classification, Tumor Differentiation, T staging, N staging, M staging, TNM staging, and PCSK9 expression were significantly associated with an increased risk of cancer-related death (**Table 2**). According to the multivariate analysis, furthermore, PCSK9 expression was an independent prognostic predictor for the GC patients with poor survival (hazard ratio [HR]: 2.158; 95% CI: 1.140–4.087; P < 0.05; **Table 2**).

**Table 2:** Univariate and multivariate analyses of clinicopathological factors for overall survival in GC patients.

Variables	Univariate analysis		Multivariate analysis	
	*P*	HR (95% CI)	*P*	HR (95% CI)
Sex (Male vs. Female)	0.598	0.899(0.606,1.334)		
Age (≤60 vs. >60)	0.955	1.012(0.665,1.541)		
Tumor size (≥5 vs. <5 cm)	0.520	1.139(0.766,1.693)		
Tumor Differentiation (Poorly vs. Well and moderately)	**0.001**	2.088(1.355,3.217)	0.374	1.339(0.703,2.548)
Lauren classification				
(Diffuse and mixed vs. Intestinal)	**0.000**	2.558(1.721,3.801)	0.129	1.554(0.880,2.743)
T stage (III+IV vs. I+II)	**0.006**	2.594(1.309,5.141)	**0.037**	2.370(1.053,5.336)
N stage (N1-3 vs. N0)	**0.000**	2.672(1.606,4.446)	**0.045**	2.123(1.018,4.429)
M stage (M1 vs. M0)	**0.000**	6.484(2.534,16.594)	**0.000**	6.605(2.455,17.771)
TNM stage (III+IV vs. I+II)	**0.001**	2.045(1.321,3.165)	0.520	0.796(0.398,1.593)
PCSK9 expression (High vs. Low)	**0.004**	2.303(1.303,4.067)	**0.018**	2.158(1.140,4.087)

HR, hazard ratio; CI, confidence interval. Bolded text shows P<0.05.

### PCSK9 Promoting Invasion and Migration and Suppressing Apoptosis of GC *In Vitro*

**Figure 2:**
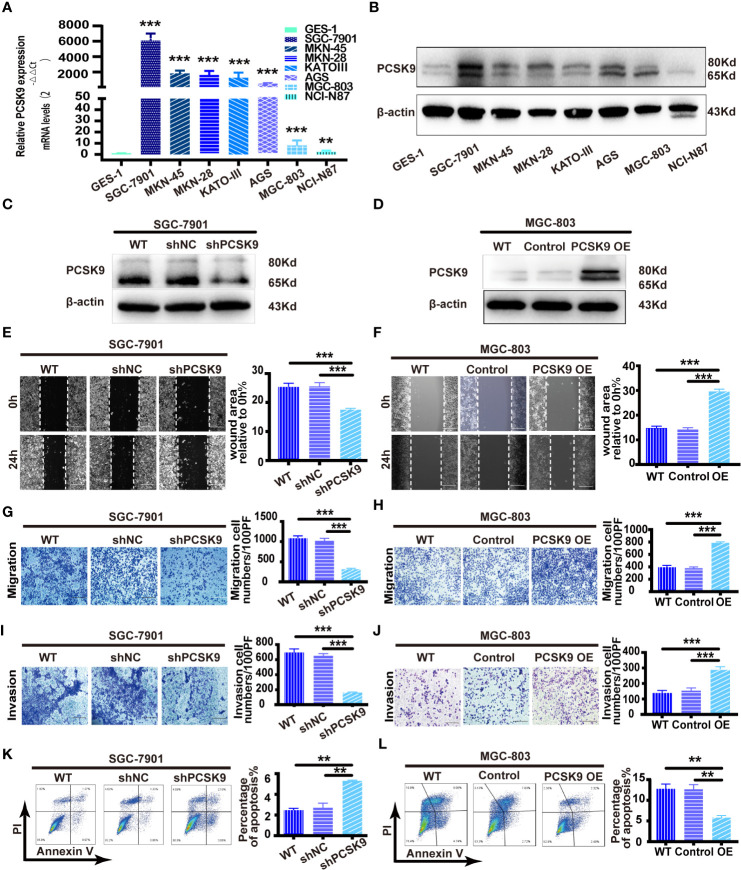
PCSK9 promoted migration and invasion and inhibited apoptosis of GC cells *in vitro*. **(A)** Relative PCSK9 expression in a panel of GC compared with GES1 cells, as determined by quantitative real-time PCR. **(B)** Western blotting of PCSK9 expression in eight cell lines; β-actin served as the internal control. **(C, D)** PCSK9 levels verified by western blotting; β-actin as the internal control. **(E)** Wound healing assay in SGC-7901 with/without PCSK9 knockdown; scale bar 100 μm. **(F)** Wound healing assay in MGC-803 cells with/without PCSK9 overexpression; scale bar 100 μm. **(G)** Transwell migration assay of SGC-7901 with/without PCSK9 knockdown; scale bar 100 μm. **(H)** Transwell migration assay of MGC-803 with/without PCSK9 overexpression; scale bar 100 μm. **(I)** Transwell invasion assay of SGC-7901 with/without PCSK9 knockdown; scale bar 100 μm. **(J)** Transwell invasion assay of MGC-803 with or without PCSK9 overexpression; scale bar 100 μm. **(K**, **L)** The cells collected and stained with FITC-Annexin V and propidium iodide; the percentage of apoptotic cells analyzed by flow cytometry assay; representative images presented. Data are expressed as mean ± S.D.; **P < 0.01, ***P < 0.001; WT, wild type; NC, negative control; OE, overexpression.

As PCSK9 expression was upregulated in GC, we hypothesized that PCSK9 could play a role in gastric cancer development. To explore the biological role of PCSK9 in GC, we applied seven human GC cell lines (SGC-7901, MKN-45, MKN-28, KATO-III, AGS, MGC-803, and NCI-N87) and a normal gastric mucosa cell line (GES-1) to the *in vivo* study. In measuring the PCSK9 mRNA and protein levels by qRT-PCR and western blotting in all these cell types, it was found that PCSK9 expression increased to varying degrees in all GC cells when compared with GES-1 (**Figures 2A, B**). Of the GC cell lines, SGC-7901 showed the highest relevant mRNA and protein level, while MGC-803 and NCI-N87 showed the lowest PCSK9 level. Accordingly, the SGC-7901 and MGC-803 cells were picked up for further experiments in view of their biological features.

Then the lentivirus of the corresponding plasmids was produced to be transduced so that PCSK9 shRNA and PCSK9 shNC in SGC-7901 and PCSK9 OE/control cells were successfully built in MGC-803 cell lines (**Figures 2C, D**). The functional experiments revealed that the wound healing rate was significantly decreased in SGC-7901 PCSK9 shRNA cells when compared with SGC-7901 PCSK9 shNC and wild type cells (P < 0.001). Meanwhile, significantly impaired migratory and invasive ability as well as increased apoptosis were found in SGC-7901 PCSK9 shRNA cells (**Figures 2E, G, I, K**). The reverse result was totally observed in PCSK9 overexpressing MGC-803 cells. Compared with the controls and wild type (WT) cells, PCSK9 OE MGC-803 cells showed enhanced healing rate, and more aggressive migration and invasion activity (**Figures 2F, H, J**). Moreover, the cell apoptosis was significantly decreased in MGC-803 cells upon PCSK9 overexpression when compared with the control group and WT cells (**Figure 2L**). However, no significant differences were observed in cell proliferation of the two cell lines and their corresponding silencing or overexpressing cells (**Supplementary Figure 1**).

### PCSK9 Up-Regulating HSP70 Expression and HSP70 Inhibition Suppressing PCSK9-Induced Invasion and Migration and Promoting Apoptosis in GC

**Figure 3:**
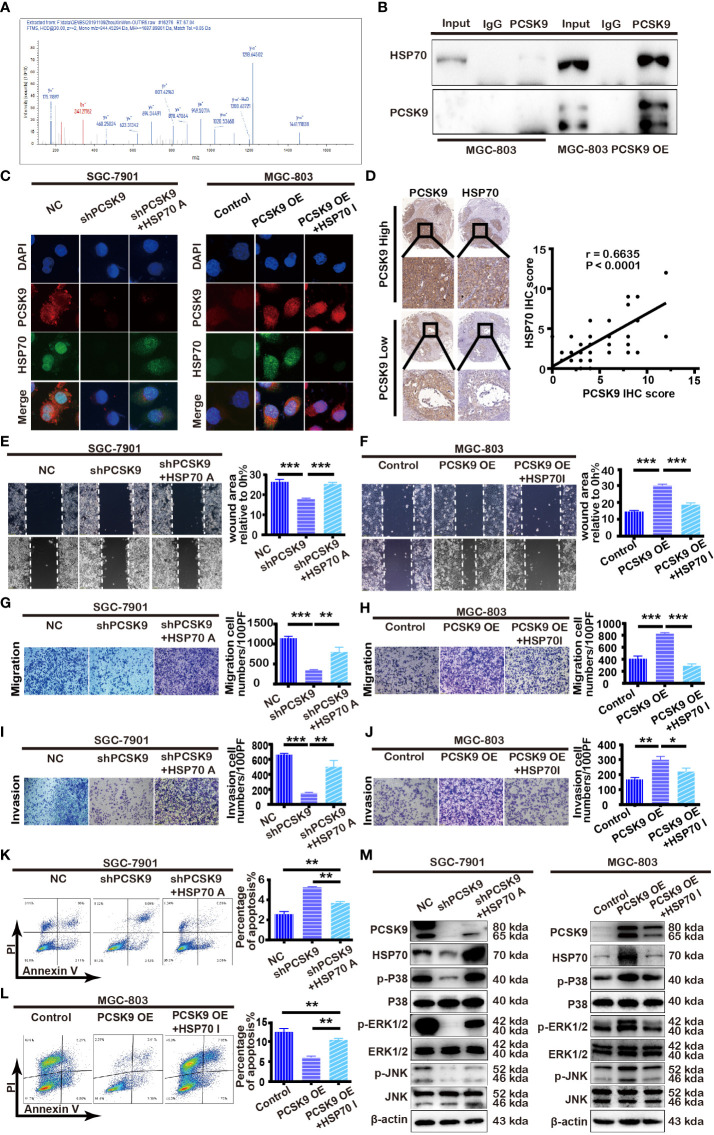
PCSK9 interacting with HSP70 and modulating MAPK pathway. **(A)** HSP70 identified by LC-MS/MS as interactor of PCSK9. **(B)** PCSK9 interacting with HSP70 in GC cell line through co-immunoprecipitation. **(C)** Confocal laser-scanning detecting the expression of PCSK9 and HSP70 in GC cells. **(D)** Correlations of PCSK9 and HSP70 protein expression in GC tissues based on immunoscoring. **(E)** The cells pretreated with TRC051384(10 μM) for 4 h; wound healing assay conducted in PCSK9 shRNA SGC-7901 cells with/without HSP70 agonist and NC cells. **(F)** The cells pretreated with apoptozole (2 μM) for 24 h; wound healing assay conducted in MGC-803 OE cells with/without apoptozole and MGC-803 control cells. **(G)** Transwell migration assay of PCSK9 shRNA SGC-7901 cells with/without HSP70 restoration and NC cells. **(H)** Transwell migration assay of MGC-803 OE cells with/without apoptozole and MGC-803 control cells. **(I)** Transwell invasion assay of PCSK9 shRNA SGC-7901 cells with/without HSP70 restoration and NC cells. **(J)** Transwell invasion assay of MGC-803 OE cells with/without apoptozole and MGC-803 control cells. **(K)** Apoptosis of SGC-7901, measured by flow cytometry. **(L)** Apoptosis of MGC-803 cells, measured by flow cytometry. **(M)** PCSK9 up-regulating HSP70 expression and activating MAPK pathway (Right: SGC-7901 cells; Left: MGC-803 cells); β-actin served as the internal control. The statistical significance between different groups was calculated with Student’s t-test; data expressed as mean ± S.D. *P < 0.05, **P < 0.01, ***P < 0.001; NC, negative control; A, agonist; I, inhibitor.

To further determine the mechanism of PCSK9 in GC, we first screened the interacting molecules with PCSK9 by co-immunoprecipitation combined with LC-MS/MS analysis, subsequently verified by immunofluorescence localization and western blotting. As seen in **Figures 3A, B**, HSP70 was screened out and verified from a total of 46 candidate protein molecules interacting with PCSK9 referring to its known function in carcinoma progression. PCSK9 and HSP 70 interacted at the cytoplasm (**Figure 3C**). Afterward, we detected the expression of HSP70 in the GC tissues by immunohistochemistry, before analyzing the relationship between the two molecules, finding that HSP70 in the cancerous tissues with high PCSK9 expression was in the state of high expression, while that with low PCSK9 expression was significantly reduced. As indicated by Pearson correlation analysis, HSP70 expression was positively correlated with PCSK9 expression (**Figure 3D**). HSP70 expression was significantly elevated in PCSK9-OE MGC-803 cells when compared with MGC-803 control cells (**Figure 3M**). The inhibition of HSP70 in PCSK9-OE MGC-803 cells suppressed cell invasion and migration while inducing increasing apoptosis (**Figures 3F, H, J, L**). Conversely, decreased HSP70 expression was detected in shPCSK9 SGC-7901 cells when compared with the controls (**Figure 3M**). The migratory and invasive ability was more enhanced in HSP70 agonist treated PCSK9 shRNA SGC-7901 cells than in non-treated PCSK9 shRNA SGC-7901cells (**Figures 3E, G, I**). Simultaneously, decreasing apoptosis was detected (**Figure 3K**). Partially because of its upregulating HSP70, PCSK9 facilitated GC cell migration and invasion and suppress apoptosis.

### PCSK9 Promoting GC Metastasis and Suppressing Apoptosis by Facilitating MAPK Signaling Pathway

Given that MAPK pathway is implicated in tumor cell proliferation, differentiation, and apoptosis, we hypothesized that it could be involved in PCSK9-induced tumor progression as well. Therefore, we monitored the phosphorylation of JNK, p38, and ERK1/2 expression level by western blotting in SGC-7901 PCSK9 knockdown cells, MGC-803 PCSK9-OE cells, and their corresponding negative controls, the results showing that the phosphorylation of JNK, p38 and ERK1/2 levels was significantly downregulated in SGC-7901 PCSK9 shRNA cells when compared with SGC-7901 PCSK9 shNC cells, and that an addition of HSP70 agonist partially restored the phosphorylation level of JNK, p38, and ERK1/2. Conversely, the protein levels were significantly upregulated in MGC-803 PCSK9-OE cells when compared with MGC-803 PCSK9 control cells. Pretreatment with apoptozole depleted MAPK activation in PCSK9-OE cells (**Figure 3M**). Thus, PCSK9 was demonstrated to play a key role in the development of GC by facilitating MAPK signaling pathway, with HSP70 acting as a synergistic role.

### PCSK9 Promoting Metastasis of GC *In Vivo*

**Figure 4:**
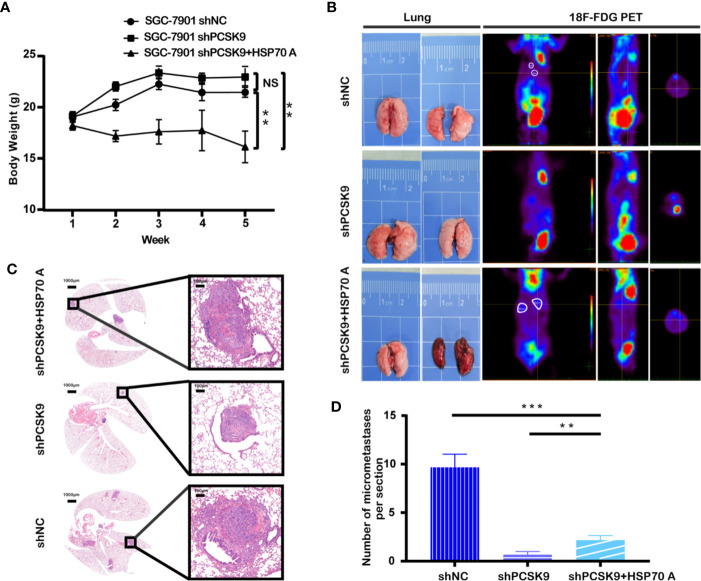
Silencing of PCSK9 suppressing the GC tumor metastasis in a mouse xenograft model and HSP70 agonist reversing this countervailing effect. **(A)** Mouse weight measured once a week at the indicated time points after injection with PCSK9 knockdown and control SGC-7901 cells until the 5^th^ week. **(B)** Representative images of lungs and PET scan of different groups (white circles represent suspected lesions). **(C)** Representative images of hematoxylin and eosin (H&E) staining of metastatic lung nodules from different groups. **(D)** Metastatic nodules in lungs of orthotopic xenograft mice model calculated (n = 10/group); data presented as mean ± S.D.; NS, not significant; **P < 0.01, ***P < 0.001.

In view of the finding that PCSK9 facilitates the migration and invasion of GC cells *in vitro*, we further tested whether PCSK9 could affect tumor progression *in vivo*. When 30 mice were equally divided into 3 groups: PCSK9 shRNA, PCSK9 shRNA+HSP70 agonist and NC group, SGC-7901cells silencing PCSK9, and its corresponding control cells were injected into the nude mice *via* the lateral tail vein. Those which bore PCSK9 shRNA SGC-7901 cells were treated with physiological saline or TRC051384 intraperitoneally for 10 days. Five weeks later, all the mice were sacrificed. As shown in **Figure 4A**, of the three PCSK9 shRNA group weighted the heaviest, whereas PCSK9 shRNA with HSP70 agonist group did the lowest (shPCSK9+HSP70 A vs. shPCSK9 vs. shNC: 16.128 ± 3.088g vs. 22.959 ± 2.717g vs. 21.459 ± 1.359 g, P < 0.001). Moreover, a trend of continued negative increase in body weight occurred in shRNA+HSP70 agonist group (5th vs. 1st: 16.128 ± 3.088 g vs. 18.210 ± 1.399 g). Knockdown of PCSK9 inhibited tumor metastasis while addition of HSP70 agonist counteracted this effect. Least metastatic lesions in lungs were found in PCSK9 silencing models through PET/CT scan and H&E staining of lung slides (**Figures 4B–D**). All this suggested that PCSK9 had an effect on promoting GC metastasis *in vivo*.

## Discussion

In this study, we explored the molecular mechanism underlying the tumor promotion of PCSK9 in GC, which, to our knowledge, represented a pioneering comprehensive analysis of PCSK9 in GC. In our cohort, PCSK9 mRNA and protein levels were significantly elevated in GC tissues when compared with paired adjacent normal tissues. The up-regulation of PCSK9 in GC could predict poor survival of GC patients, highlighting the potential of PCSK9 as a novel biomarker for GC.

A growing amount of evidence has manifested that PCSK9 show a promising advantage to cancer progression as well as poor clinical prognosis in several malignant tumors, such as melanoma (14), non-small cell lung cancer (NSCLC) (15), breast cancer (16), and hepatocellular carcinoma(HCC) (20). Our findings demonstrated that high PCSK9 expression was correlated with lymph node metastasis and that it is the independent predictor for GC patients with poor survival. This may be attributed to its internal ability in both cell proliferation and apoptosis. Under physiological conditions, PCSK9 is mainly expressed in the liver and, to a lesser extent, in the pancreas, kidney, brain and small intestine but hardly expressed in the stomach (21). PCSK9 was initially recognized as neural apoptosis regulated convertase1 (NARC1) (22), which represents the molecule in brain apoptosis. In the models of mice that underwent partial hepatectomy, PCSK9 expression was highly induced in the liver, even if under a PCSK9 defect genetic background, a high-cholesterol diet could rescue liver cell proliferation (22, 23). In our study, we found that PCSK9 expression was upregulated in GC cells, which significantly inhibited cell apoptosis and promoted cell invasion and migration as well. However, it didn’t have an effect on proliferation in GC cell lines. Apart from the features of uncontrollable proliferation and apoptotic resistance in tumor cells, invasion and migration have been recognized as the most two important hallmarks leading to the lethality in GC (24, 25). As indicated by the current results, PCSK9 depletion in SGC-7901 cells distinctly suppressed the migrative and invasive ability and upregulated apoptotic rate when compared with the cells transduced with the empty vector, and the inverse results were found in PCSK9 overexpression MGC-803 cells in comparison with the MGC-803 controls. Our *in vivo* experiments were also consistent with the *in vitro* data that PCSK9 knockdown in SGC-7901 cells significantly inhibited tumor metastasis in the lungs, as indicated by the model of xenograft nude mice. The significant correlation between the PCSK9 expression and migration/invasion and apoptosis of GC suggested that PCSK9 could serve as a “metastasis promotor gene” in GC.

Subsequently, we managed to pick up HSP70 as the interacting molecule in PCSK9 tumor regulation, which played a synergistic role in the process. HSP70, a member of the evolutionarily highly conserved heat shock protein family, plays an important role in enhancing cell resistance to various stimuli and alleviating cell damage *via* its molecular chaperone function in protein folding, unfolding, and disaggregation (26). Lines of studies have revealed that the basal level of HSP70 was high in several types of malignancies (27–31) and that it has been categorized as a bad prognosis factor (27, 28). HSP70 has also been demonstrated to cooperate in chemotherapy and radiotherapy resistance probably due to its capacity to disable cell death (32–34). Consistent with these pieces of evidence, our findings indicated that HSP70 had a high expression in GC tissues when compared with the adjacent normal tissues. High expression of HSP70 is known to be in accordance with the high expression level of PCSK9. In parallel, the tissues with low expression of PCSK9 showed a consistent low expression of HSP70. *In vitro*, HSP70 rarely expressed in PCSK9 depleted SGC-7901 cells, but highly expressed in PCSK9 overexpression MGC-803 cells. The expression of the two molecules had a positive correlation. Inducing HSP70 expression could reverse the inhibition effect of silencing PCSK9 in SGC-7901 both *in vitro* and *in vivo*. Thereby, PCSK9 up-regulation can be responsible for the induction of HSP70 expression in metastatic GC, which promotes the migration and invasion of GC.

Furthermore, we found that MAPK signaling pathway was involved in the PCSK9-induced cancer promotion and that the silencing of PCSK9 impeded the phosphorylation of p38 MAPK, ERK1/2, and JNK, thus significantly downregulating the migration and invasion of cells. HSP70 induction could partially transverse this effect. Conversely, the overexpression of PCSK9 in MGC-803 cells facilitated MAPK signaling pathway, accelerating the migration/invasion of the cells when compared with the controls. Previous studies have shown that MAPK signaling pathway plays a central role in the stimulation of cancer cell proliferation, apoptosis, and metastasis (35–37). And also p38 MAPK participates in resistance to chemotherapy drugs like cisplatin, irinotecan, and 5-fluorouracil (38), which are often administered in GC chemotherapy regimens. However, Bode AM et al. (39) found an antiapoptotic effect of JNK pathway in GC, which seemed to be controversial. Our results showed that p38, ERK1/2, and JNK were all activated in the PCSK9-related cancer procedure, and that the modulation of JNK showed a pro-apoptotic effect.

It is undeniable that there are some limitations to our study. We detected PCSK9 expression in 155 GC tissues and serum PCSK9 protein levels preoperatively in 60 patients. The sample size is relatively small. Besides, the follow-up time of the cohort is partially short and we failed to achieve the 5-year overall survival time. Thus, additional studies with larger sample sizes, longer follow-up periods are needed to confirm our findings.

In recent years, the link between cholesterol metabolism and cancer progression has drawn a great deal of attention. Of note, PCSK9, which stands for the up-regulation of LDL cholesterol *via* promoting LDL receptor degrading, may participate in cancer progression by modulating cholesterol supply to the tumor, as indicated in a B cell tumor mouse model in which hepatic PCSK9 expression and secretion were induced, leading to hepatic LDLR reduction and hypercholesterolemia. More exogenous lipids were transported to the tumor cells to support the genesis and proliferation of the tumor (40). Abdelwahed KS et al. (16) suggested that suppression of the PCSK9-LDLR axis could inhibit tumor progression and recurrence in the hormone-dependent breast cancer in a nude mouse xenograft model. Interestingly, HSP70 was also found upregulated in the livers of obese mice (41). HSP70 overexpression in HepG2 cells enhanced the synthesis of cholesterol, the size of lipid droplets increasing simultaneously. Moreover, it was indicated that LDL enhances colorectal cancer progression *via* MAPK signaling pathway (42). In our study, we showed that PCSK9 could promote tumor metastasis in GC partly through HSP70 up-regulation by modulating MAPK pathway. This exciting finding prompted us to pursue further studies to reveal a profound understanding of PCSK9 in tumor promotion, especially in lipid metabolism.

## Conclusion

To conclude, we validated in our study that PCSK9 could function as a deleterious biomarker in GC; that high PCSK9 expression levels in GC tissue could be correlated with GC progression and prognosis; and that PCSK9 could promote GC metastasis and suppress apoptosis *via* facilitating MAPK signaling pathway through HSP70 up-regulation. Additionally, we identified PCSK9 as a harmful molecule in tumor progression, which indicates that the inhibition of PCSK9 can be a novel and promising therapeutic approach to GC.

## Data Availability Statement

The raw data supporting the conclusions of this article will be made available by the authors, without undue reservation.

## Ethics Statement

The studies involving human participants were reviewed and approved by the Clinical Research Ethics Committee of Fudan University. The patients/participants provided their written informed consent to participate in this study. The animal study was reviewed and approved by the Animal Ethics Committee of Zhongshan Hospital.

## Author Contributions

BX, SL, YF, and YC wrote the manuscript. BX and SL executed the experiments. BX, SL, and YF collected clinicopathological data and GC specimens. BX, SL, and YZ analyzed the data. DS added support to the making of the figures and tables. YC and SZ dominated in designing the study, instructing the experiments, and reviewing the manuscript. All authors contributed to the article and approved the submitted version.

## Funding

This work was supported by the Shanghai Science and Technology Commission (grant no. 19ZR1409400).

## Conflict of Interest

The authors declare that the research was conducted in the absence of any commercial or financial relationships that could be construed as a potential conflict of interest.
