# Supplementary figures and images for: Proprotein Convertase Subtilisin/Kexin Type 9 Promotes Gastric Cancer Metastasis and Suppresses Apoptosis by Facilitating MAPK Signaling Pathway Through HSP70 Up-Regulation
Source: Front Oncol. 2021 Jan 7;10:609663. doi: 10.3389/fonc.2020.609663 (PMC7817950; doi:10.3389/fonc.2020.609663)

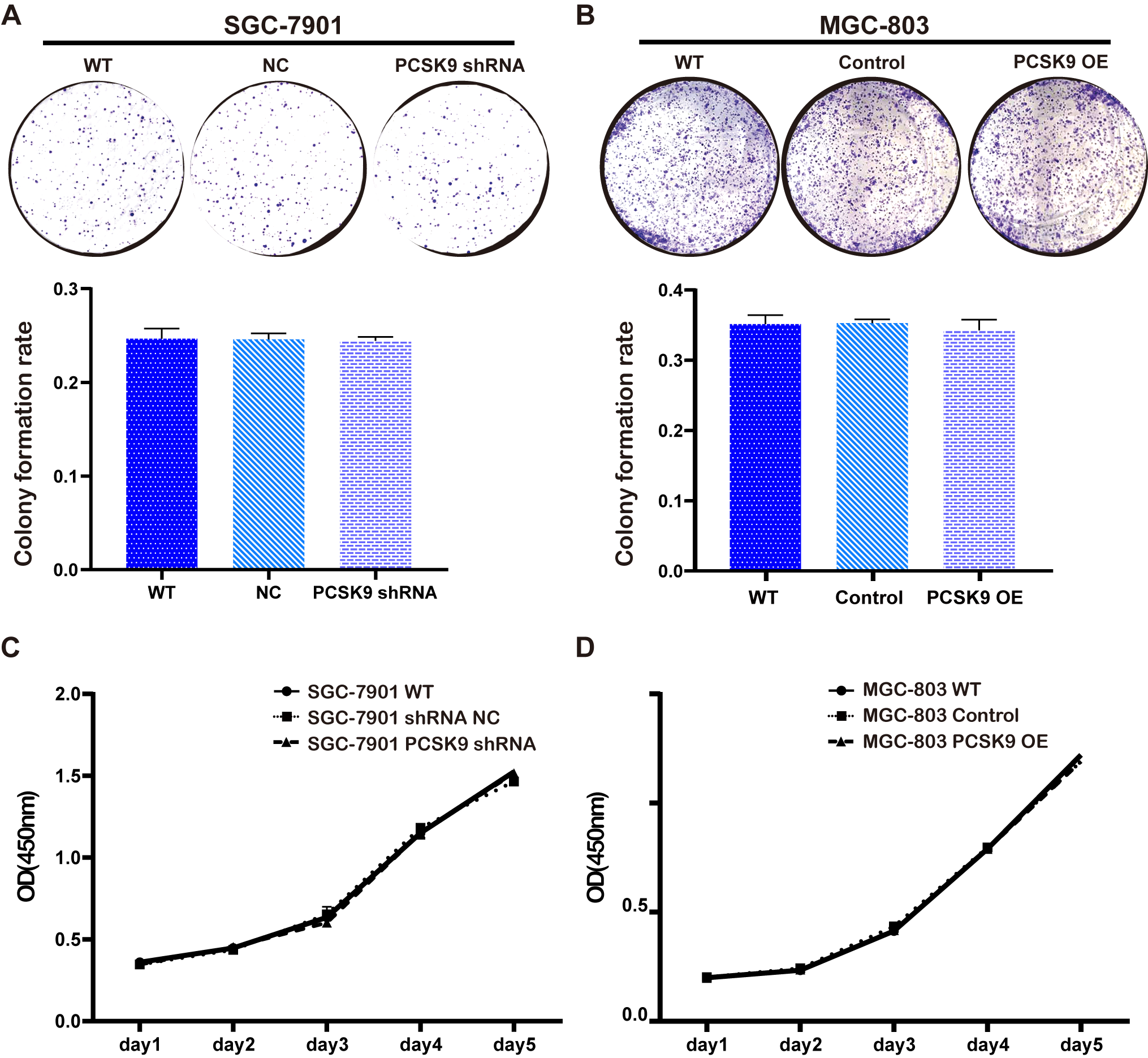

Supplement: Supplementary file 1 [file Image_1.tif]
