# Supplementary material for: Proprotein Convertase Subtilisin/Kexin Type 9 Promotes Gastric Cancer Metastasis and Suppresses Apoptosis by Facilitating MAPK Signaling Pathway Through HSP70 Up-Regulation
Source: Front Oncol. 2021 Jan 7;10:609663. doi: 10.3389/fonc.2020.609663 (PMC7817950; doi:10.3389/fonc.2020.609663)
Supplement: Supplementary file 2 [file Table_1.docx]

**Supplementary Table 1**. Clinical data of the gastric cancer patients

|  | N=155 |
| --- | --- |
| Sex |  |
| Male | 100 |
| Female | 55 |
| Age(y) |  |
| ≤60 | 45 |
| >60 | 110 |
| Tumor differentiation |  |
| Well | 2 |
| Moderately | 50 |
| Poorly | 103 |
| Tumor size(cm) |  |
| <5 | 80 |
| ≥5 | 75 |
| Lauren type |  |
| Intestinal  Diffused and mixed | 72  83 |
| T stage |  |
| I-II | 20 |
| III-IV | 135 |
| Lymph node metastasis |  |
| No | 38 |
| Yes | 117 |
| M stage |  |
| M0 | 150 |
| M1 | 5 |
| liver | 2 |
| peritoneum | 2 |
| ovary | 1 |
| TNM stage |  |
| I+II | 49 |
| III+IV | 106 |

**SUPPLEMENTARY FIGURE 1.** PCSK9 has no effect on GC cell proliferation. (A) Clonogenic assay in SGC-7901 cells with/without PCSK9 silencing. (B) Clonogenic assay in MGC-803 PCSK9 cells with/without PCSK9 overexpression. (C) Cell growth curves of SGC-7901 cells using CCK-8 assay. (D) Cell growth curves of MGC-803 cells using CCK-8 assay.
